# FC-TRIPLEX Chagas/Leish IgG1: A Multiplexed Flow Cytometry Method for Differential Serological Diagnosis of Chagas Disease and Leishmaniasis

**DOI:** 10.1371/journal.pone.0122938

**Published:** 2015-04-15

**Authors:** Andréa Teixeira-Carvalho, Fernanda Magalhães Freire Campos, Stefan Michael Geiger, Roberta Dias Rodrigues Rocha, Fernanda Fortes de Araújo, Danielle Marquete Vitelli-Avelar, Mariléia Chaves Andrade, Márcio Sobreira Silva Araújo, Elenice Moreira Lemos, Anna Bárbara de Freitas Carneiro Proietti, Ester Cerdeira Sabino, Rafaella Gaiotti Caldas, Carolina Renata Camargos Freitas, Ana Carolina Campi-Azevedo, Silvana Maria Elói-Santos, Olindo Assis Martins-Filho

**Affiliations:** 1 Laboratório de Biomarcadores de Diagnóstico e Monitoração, Centro de Pesquisas René Rachou, Fundação Oswaldo Cruz, Belo Horizonte, Minas Gerais, Brasil; 2 Laboratório de Helmintoses Intestinais, Departamento de Parasitologia, Instituto de Ciências Biológicas, Universidade Federal de Minas Gerais, Belo Horizonte, Minas Gerais, Brasil; 3 Departamento de Fisiopatologia, Centro de Ciências Biológicas e da Saúde, Universidade Estadual de Montes Claros, Montes Claros, Brasil; 4 Núcleo de Doenças Infecciosas, Centro Biomédico, Universidade Federal do Espírito Santo, Vitória, Brasil; 5 Serviço de Pesquisa, Fundação Centro de Hematologia e Hemoterapia de Minas Gerais—HEMOMINAS, Belo Horizonte, Minas Gerais, Brasil; 6 Departamento de Doenças Infecciosas, Instituto de Medicina Tropical, Universidade de São Paulo—USP, São Paulo, Brasil; 7 Departamento de Pós-Graduação em Patologia Geral, Faculdade de Medicina, Universidade Federal de Minas Gerais, Belo Horizonte, Minas Gerais, Brasil; 8 Departamento de Propedêutica Complementar, Faculdade de Medicina, Universidade Federal de Minas Gerais, Belo Horizonte, Minas Gerais, Brasil; Federal University of São Paulo, BRAZIL

## Abstract

Differential serological diagnosis of Chagas disease and leishmaniasis is difficult owing to cross-reactivity resulting from the fact that the parasites that cause these pathologies share antigenic epitopes. Even with optimized serological assays that use parasite-specific recombinant antigens, inconclusive test results continue to be a problem. Therefore, new serological tests with high sensitivity and specificity are needed. In the present work, we developed and evaluated the performance of a new flow cytometric serological method, referred to as FC-TRIPLEX Chagas/Leish IgG1, for the all-in-one classification of inconclusive tests. The method uses antigens for the detection of visceral leishmaniasis, localized cutaneous leishmaniasis, and Chagas disease and is based on an inverted detuned algorithm for analysis of anti-Trypanosomatidae IgG1 reactivity. First, parasites were label with fluorescein isothiocyanate or Alexa Fluor 647 at various concentrations. Then serum samples were serially diluted, the dilutions were incubated with suspensions of mixed labeled parasites, and flow cytometric measurements were performed to determine percentages of positive fluorescent parasites. Using the new method, we obtained correct results for 76 of 80 analyzed serum samples (95% overall performance), underscoring the outstanding performance of the method. Moreover, we found that the fluorescently labeled parasite suspensions were stable during storage at room temperature, 4°C, and –20°C for 1 year. In addition, two different lots of parasite suspensions showed equivalent antigen recognition; that is, the two lots showed equivalent categorical segregation of anti-Trypanosomatidae IgG1 reactivity at selected serum dilutions. In conclusion, we have developed a sensitive and selective method for differential diagnosis of Chagas disease, visceral leishmaniasis, and localized cutaneous leishmaniasis.

## Introduction

Chagas disease (CH) is thought to affect 16–18 million people worldwide, and the number of infected individuals in the main areas of transmission in Latin America is estimated to be 12–14 million [1]. Because in areas where efficient control of the triatomine bugs that transmit CH has been achieved, local ministries of health have paid more attention to transmission via contaminated food, vertical transmission *in utero*, and transmission by blood transfusions and organ transplantation [2]. For example, the high risk of CH infection via blood transfusion led to mandatory serological screening of blood donors in Latin American countries, and as a result, the risk of this transmission route has been reduced [3, 4]. However, preventing new infections via blood transfusion remains of utmost importance, both in Latin American countries and in countries with many immigrants from Latin America [5].

During the chronic phase of *Trypanosoma cruzi* infection, the number of parasites in the peripheral blood is small, and therefore isolation and detection of the parasite is difficult. Because of the low sensitivity of parasitological exams, CH is usually diagnosed by means of various indirect serological methods, such as enzyme-linked immunosorbent assay (ELISA), indirect hemagglutination assay, indirect immunofluorescence assay, western blotting, and immunochromatography [6], and positive results for at least two different serological tests are required for a conclusive result [7]. At present, one specific screening test for CH is conducted on blood donations in Brazil, and if the result is inconclusive, a different, more-specific test is used [8].

Depending on the antigens used, serological tests show cross-reactivity with other infectious agents, e.g., *Leishmania* spp. and other trypanosomatids [5]; and approximately 3–5% of blood donations show an indeterminate result; that is, only one test shows reactivity [9]. Even with optimized serological assays that use parasite-specific recombinant antigens, inconclusive test results continue to be a problem [8, 10]. The fact that some infected individuals show repeated inconclusive results and low-level seroreactivity causes considerable concern in blood centers in Brazil, with regard to both blood donor counseling and CH epidemiology [8]. Therefore, the search for new immunodominant antigens and serological tests with high sensitivity and specificity continues [11], and various algorithms for more-precise identification of truly infected patients have been proposed [8].

Our research group has introduced the use of flow cytometry (FC) in combination with fixed parasite material as an inexpensive, reliable technique for detecting and classifying American tegumentary leishmaniasis [12] and visceral leishmaniasis (VL) [13], as well as various clinical forms of CH [14–16]. Here, we report the development and performance of a new FC-based serological method, referred to as FC-TRIPLEX Chagas/Leish IgG1, for all-in-one classification of inconclusive Chagas tests; the method uses antigens for the detection of VL, localized cutaneous leishmaniasis (LCL), and CH.

## Population, Materials, and Methods

### Study Population

A total of 80 human serum samples were obtained from our biorepository and divided into the following four groups: (1) 20 serum samples from healthy blood donors with negative serology for common infectious and parasitic diseases (non-infected controls, NI); (2) 20 serum samples from patients with CH, as indicated by positive xenodiagnosis and two positive serological tests (ELISA and immunofluorescence assay); (3) 20 serum samples from patients with LCL, as indicated by positive results for both Montenegro's skin test and an immunofluorescence assay; and (4) 20 serum samples from patients with VL, as indicated by positive direct parasite detection and positive serological tests (ELISA and immunofluorescence assay). All samples were provided from adult individuals with age ranging between 18 to 55 years old.

### Ethics Statements

This study was approved by the Ethical Committee at the HEMOMINAS Foundation (no. 157/2007) and by the Ethical Committee at René Rachou Research Center, Oswaldo Cruz Foundation (protocol no. CAAE: 34644614.0.0000.5091). The study was conducted according to the Brazilian national guidelines for research with human subjects (resolution no. 466/2012). Informed written consent was obtained from all participants before enrollment in the study.

### Sample Processing

The serum samples were inactivated at 56°C for 30 min and then centrifuged at 14,462 x *g* at 4°C for 5 min to remove particles. After centrifugation, an aliquot of the supernatant was removed and stored at -20°C until use in the FC assays. Prior to the assays, the stored serum samples were thawed, diluted in phosphate buffered saline (PBS) supplemented with 3% fetal calf serum (Sigma, St. Louis, MO, USA), and centrifuged at 14,462 x *g* for 5 min at 4°C.

### Parasite Preparations

Epimastigotes of *T*. *cruzi* were obtained from liquid parasite cultures in complex liver infusion tryptose (LIT) and were incubated in an environmental chamber at 28 ± 1°C, according to previously described procedures [14]. Every 7 days, the cultures were spiked with 1.0 x 10^6^ parasites/mL, and the cultures were maintained by successive passages in LIT medium. Before use in the FC assays, parasites were fixed in paraformaldehyde, and the parasite concentration was adjusted to 1 x 10^7^ parasites/mL [14].

Promastigotes of *L*. *braziliensis* (MHOM/BR/75/M2903 strain) and *L*. *infantum* (MHOM/BR/74/PP75 strain) were cultivated in Novy-MacNeal-Nicolle (NNN) blood agar associated with LIT medium (NNN-LIT) and incubated at 24 ± 1°C, according to previously described procedures [12,13]. Every 2 days, the cultures were spiked with 5.0 x 10^6^ parasites/mL in medium NNN associated with M199 medium (NNN-M199), and the cultures were maintained by successive passages in NNN-M199. Parasites were collected at the stationary growth phase, and centrifuged at 100 x *g* for 10 min at room temperature to remove cell debris and erythrocytes. The supernatant containing the parasites was centrifuged at 1,000 x g for 10 min, 4°C.

### Preparation of *T*. *cruzi* Epimastigotes and *L*. *braziliensis* and *L*. *infantum* Promastigotes for Immunofluorescence Assay Using FC

Epimastigotes of *T*. *cruzi* that had been cultivated for 7 days in LIT medium [14] and *L*. *braziliensis* and *L*. *infantum* promastigotes that had been cultivated for 4 days in NNN-M199 medium were transferred separately to three 50-mL polypropylene tubes, and homogenized in a vortex at low speed (speed 3) to break up the lumps. The suspensions were differentially centrifuged at 200 x *g* for 10 min at 25°C to remove dead parasites in the sediment. For recovery of living parasites in the supernatant, the tubes were left to stand for 30 min at room temperature. The supernatants were transferred to other 50-mL polypropylene tubes, and the pellets were discarded. The parasites were then washed twice with PBS and centrifuged at 1000 x *g* for 10 min at 4°C, and the pellets were carefully homogenized and resuspended in PBS. The suspensions were adjusted to parasite concentrations of 1.0 x 10^7^ parasites/mL in PBS and were fixed in fluorescence-activated cell sorting fix solution (paraformaldehyde, 10 g/L; 1% sodium cacodylate; NaCl, 6.65 g/L; 0.01% sodium azide; pH 7.2).

### Differential Staining of *T*. *cruzi* Epimastigotes and *L*. *braziliensis* and *L*. *infantum* Promastigotes With Fluorescein Isothiocyanate and Alexa Fluor 647

**Fig 1:**
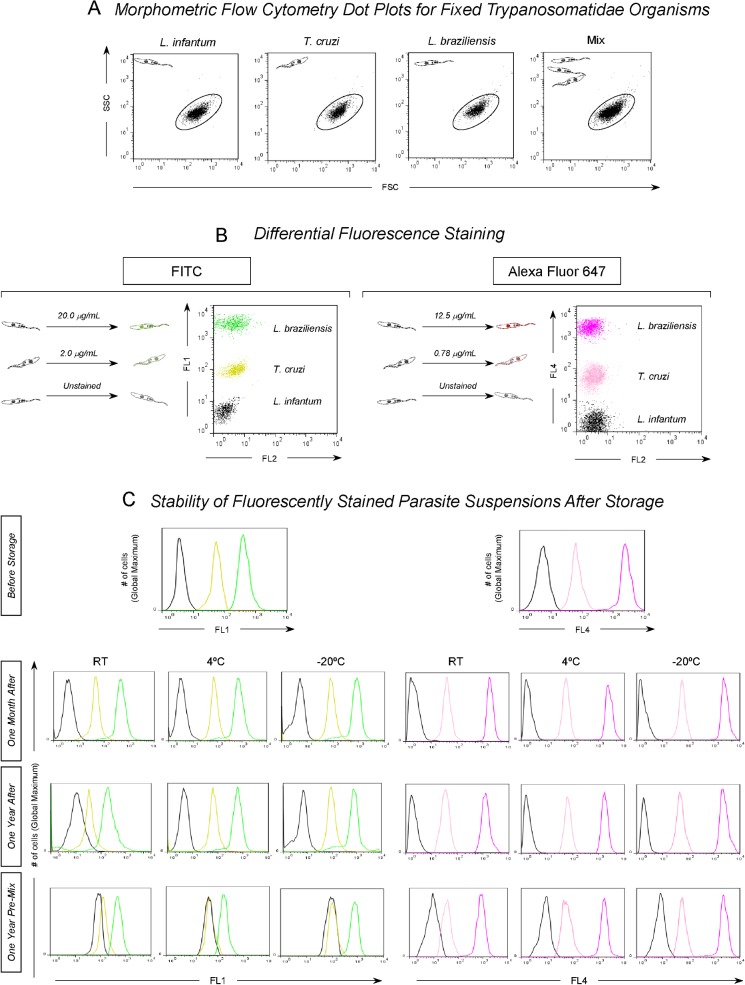
Flow cytometry-based triplex array (FC-TRIPLEX) for selective analyses of *L*. *infantum* promastigotes, *T*. *cruzi* epimastigotes, and *L*. *braziliensis* promastigotes. (A) Representative dot plots demonstrating similarities in the morphometric features of the fixed Trypanosomatidae organisms. (B) Differential fluorescence staining with fluorescein isothiocyanate (FITC) (*L*. *infantum* = 0.0 μg/mL, *T*. *cruzi* = 2.0 μg/mL, or *L*. *braziliensis* = 20.0 μg/mL) or Alexa Fluor 647 (*L*. *infantum* = 0.0 μg/mL, *T*. *cruzi* = 0.78 μg/mL, or *L*. *braziliensis* = 12.5 μg/mL) as a strategy for segregating parasites based on the FL1 and FL4 fluorometric profiles, respectively. (C) Unidimensional histograms for the FL1 and FL4 profiles illustrating the stability of fluorescently stained single or premixed parasite suspensions during storage at room temperature (RT), 4°C, or −20°C.

Parasite suspensions (1.0 x 10^7^ parasites/mL) were incubated with various concentrations of fluorescein isothiocyanate (FITC) or Alexa Fluor 647 for 30 min at 37°C in the dark. After incubation, the parasites were washed with PBS containing 10% fetal bovine serum (PBS-FBS) and then centrifuged at 1,000 x *g* for 10 min at 4°C. The supernatants were discarded, the pellet was carefully homogenized and resuspended in PBS, and the suspensions were adjusted to parasite concentrations of 5.0 x 10^6^ parasites/mL before use in the FC assays (Fig 1).

### Flow Cytometric Immunofluorescence Assay

**Fig 2:**
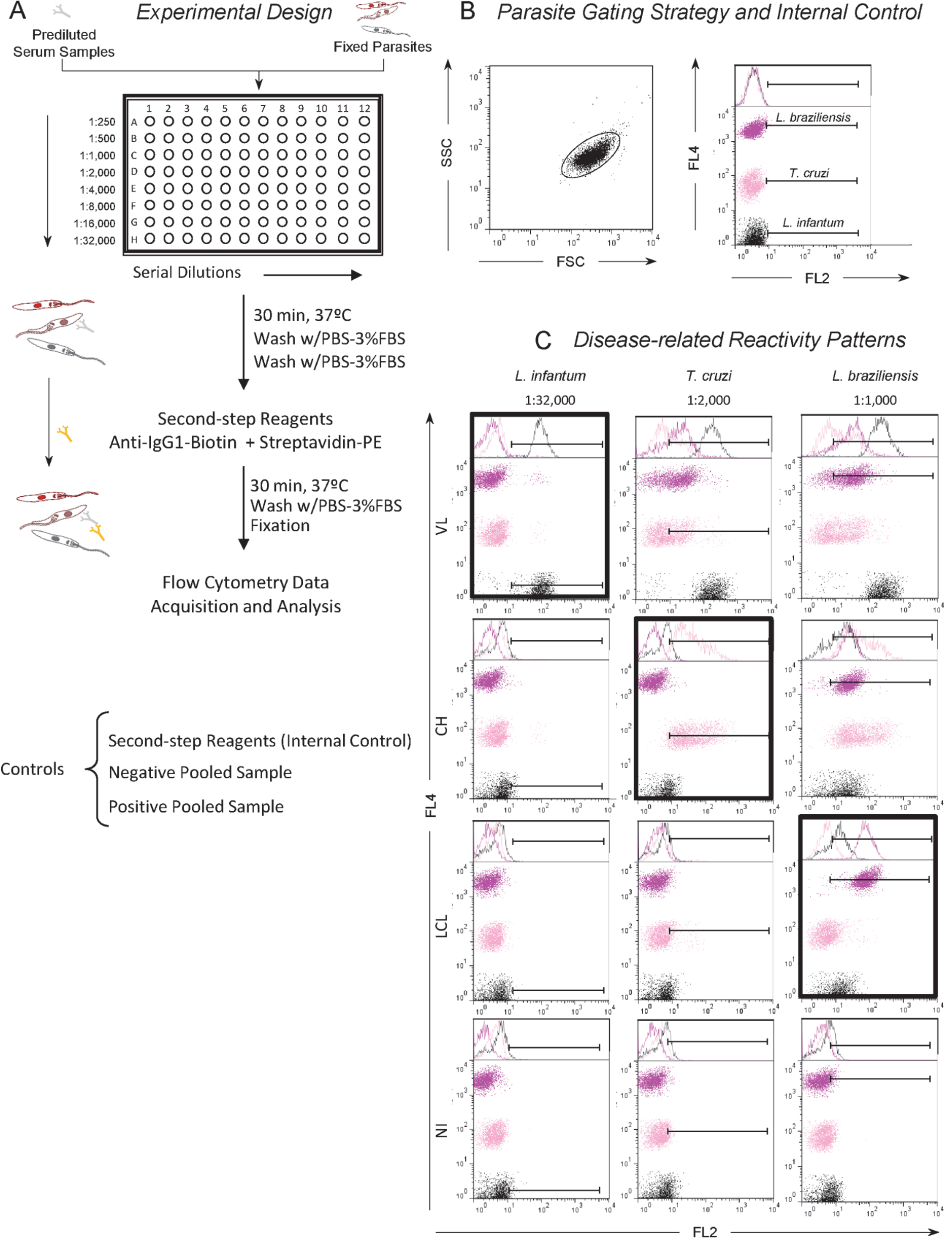
Experimental design for simultaneous detection of anti-Trypanosomatidae IgG1 antibodies by flow cytometry-based triplex array (FC-TRIPLEX Chagas/Leish IgG1). (A) Major assay steps. PBS, phosphate-buffered saline; FBS, fetal bovine serum; PE, phycoerythrin. (B) Parasite gating strategy based on FCS versus SSC dot plot distribution followed by a set marker setup for internal control positivity limit, leading to a Percentage of Positive Fluorescent Parasites value of <2.0%. (C) This set marker setup was maintained to subsequently determine disease-related reactivity patterns. VL, visceral leishmaniasis; CH, Chagas disease; LCL, localized cutaneous leishmaniasis; NI, noninfected controls. Dark frames highlight species-specific IgG1 reactivity patterns at selected serum dilutions (VL, anti-*L*. *infantum* at 1:32,000; CH, anti-*T*. *cruzi* at 1:2,000; LCL and NI, anti-*L*. *braziliensis* at 1:1,000) according to Garcia et al. [13], Matos et al. [15], and Pereira et al. [12], respectively.

The immunofluorescence assay was performed as reported by Martins-Filho et al. [17] with modifications described by Lemos et al. [18]. Briefly, fluorescent trypanosomatids (50 μL/well), 150,000 fixed *L*. *infantum* promastigotes, 150,000 fixed *T*. *cruzi* epimastigotes, and 150,000 fixed *L*. *braziliensis* differentially stained with FITC (*L*. *infantum* = 0.0 μg/mL, *T*. *cruzi* = 2.0 μg/mL, or *L*. *braziliensis* = 20.0 μg/mL) or Alexa Fluor 647 (*L*. *infantum* = 0.0 μg/mL, *T*. *cruzi* = 0.78 μg/mL, or *L*. *braziliensis* = 12.5 μg/mL) were incubated for 30 min at 37°C in the presence of 50 μL of serial dilutions (1:250 to 1:32,000) of individual sera from VL, CH, and LCL patients and from healthy controls-NI (Fig 2A). After incubation, the parasites were washed twice with 150 μL of PBS containing 3% FBS at 1000 x *g* for 10 min at 4°C, re-incubated at 37°C for 30 min in the dark in the presence of 50 μL of biotinylated anti-human IgG1 antibody (Sigma), and diluted 1:6,400 in PBS containing 3% FBS. The detecting antibody was then incubated in the presence of 20 μL of streptavidin conjugated to phycoerythrin at a 1:400 dilution in PBS containing 3% FBS. After a second washing step, the parasites labeled with FITC or Alexa Fluor 647 were fixed for 30 min with 200 μL of fluorescence-activated cell sorting fix solution before analysis in the cytometer. Samples not read immediately were kept at 4°C in the dark. The maximum delay in data acquisition was 24 h. Each assay included an internal control for nonspecific binding, in which fixed parasites that had not been exposed to human serum were incubated with phycoerythrin-conjugated anti-human IgG1 and subjected to the same procedures as the serum-exposed parasites (Fig 2A).

### Fluorescence-Activated Cell Sorting Data Storage and Analysis

FC was performed on a Becton Dickinson FACSort interfaced to an Apple Quadra FACStation (FACSCalibur, Becton Dickinson). The CellQuest software package (Becton Dickinson) was used for data storage, and data were analyzed with FlowJo software. Stained parasites exposed to the different serum samples were run in the cytometer (FACSCalibur), and 10,000 events per sample were acquired.

### Statistical Analysis

A receiver operating characteristic (ROC) curve was constructed by plotting sensitivity on the *y*-axis and the complement of specificity (100 − specificity) on the *x*-axis. The curve was used to select the cut-off value to determine percentages of positive fluorescent parasites (PPFP). The global accuracy of the method was evaluated by determining the area under the ROC curve. The performance of each run of the assay was assessed by means of three statistical indexes: (1) sensitivity = [true positives/(true positives + false negatives)] × 100; (2) specificity = [true negatives/(true negatives + false positives)] × 100; and (3) likelihood ratios (LR), where the LR for positive results (LR+) = [sensitivity/(1—specificity)] and the LR for negative results (LR−) = [(1 − sensitivity)/specificity]. LR+ values of >10 practically confirmed disease diagnosis, whereas LR values of < 0.1 practically excluded disease diagnosis, as previously proposed [19]. All statistical analyses were performed by using the MedCalc software package (ver. 7.3).

## Results

### FC-Based Triplex Labeling Array (FC-TRIPLEX) for Selective Analyses of *L*. *infantum* Promastigotes, *T*. *cruzi* Epimastigotes, and *L*. *braziliensis* Promastigotes

Because the *T*. *cruzi* epimastigote and *L*. *braziliensis* and *L*. *infantum* promastigote populations have similar morphometric features (and thus similar forward scattering [FSC] and side scattering [SSC]; Fig 1A), we used differential fluorescence staining with FITC or Alexa Fluor 647 to segregate the parasites based on the fluorometric profiles of FL1 and FL4, respectively (Fig 1B). In the dot plots for parasite staining with FITC or Alexa Fluor 647, the *y*-axis shows the fluorescence intensity of FL1 or FL4, respectively, and the *x*-axis represents the intensity of secondary staining (FL2). All the parasites in each mixture were discriminated on the basis of the intensity of the first emitted fluorescence. The parasites that showed high fluorescence intensity corresponded to *L*. *braziliensis* promastigotes, allowing us to gate on the corresponding region in this population (R2). Parasites with mean fluorescence intensity corresponded to *T*. *cruzi* epimastigotes, allowing us to gate on the corresponding region in this population (R3). Finally, nonfluorescent parasites corresponded to *L*. *infantum* promastigotes (gate R4) (Fig 1B).

For evaluation of the stability of the parasite suspensions, suspensions of individual parasites or premixed parasites stained with FITC or Alexa Fluor 647 were stored at room temperature, 4°C, or—20°C for 1 year, and the fluorescence intensity of the suspensions was assessed monthly by FC. We found that the suspensions of stained individual parasites and premixed parasites remained stable for 1 year at all three temperatures evaluated. However, the premixed labeled parasite suspensions showed considerable overlap after 1 year of storage under the described conditions (Fig 1C).

### Experimental Design for Simultaneous Detection of Anti-Trypanosomatidae IgG1 Antibody by FC-TRIPLEX Chagas/Leish IgG1

Serum samples were serially diluted and incubated with mixed parasite suspensions stained with Alexa Fluor 647 as described in Population, Materials, and Methods (Fig 2A). For each mixed parasite suspension, *L*. *infantum*, *T*. *cruzi*, and *L*. *braziliensis* could be differentiated on the basis of the fluorescence intensity of FL4 (Fig 2B). Binding of serum antibodies to the differentially stained parasite populations and shift of each population by detection of secondary phycoerythrin-stained antibodies (as relative fluorescence intensity in FL2) was analyzed in a single histogram representation (Fig 2B and 2C). For sequential analysis, an algorithm was introduced in which serum samples were analyzed stepwise at serum dilutions of 1:32,000 for VL, 1:2,000 for CH, and 1:1,000 for LCL (Fig 2C).

### Species-Specific Anti-Trypanosomatidae IgG1 Reactivity and FC-TRIPLEX Chagas/Leish IgG1 ROC Curve Performance Indexes

**Fig 3:**
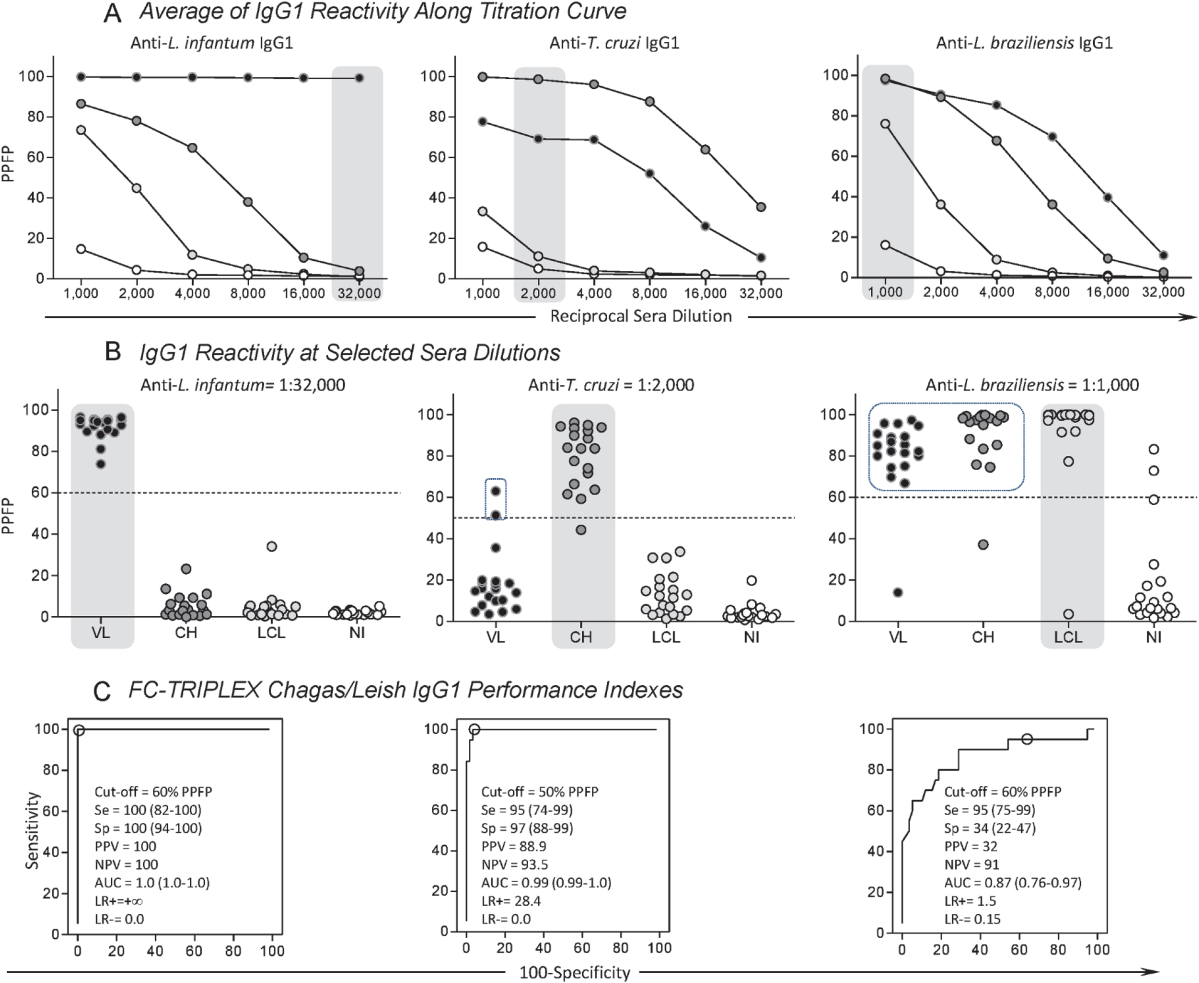
Species-specific anti-Trypanosomatidae IgG1 reactivity and flow cytometry-based triplex array (FC-TRIPLEX Chagas/Leish IgG1) performance indexes. (A) Titration curve averages of percentages of positive fluorescent parasites (PPFP) for anti-Trypanosomatidae IgG1 in serum samples from patients with visceral leishmaniasis (VL, black circles), patients with Chagas disease (CH, dark gray circles), localized cutaneous leishmaniasis (LCL, light gray circles), and noninfected controls (NI, white circles). (B) Scattering plots for individuals at selected sera dilutions highlighted by gray round rectangles (VL, anti-*L*. *infantum* at 1:32,000; CH, anti-*T*. *cruzi* at 1:2,000; LCL and NI, anti-*L*. *braziliensis* at 1:1,000) using the cut-off edges (dotted lines), previously described by Garcia et al. [13], Matos et al. [15], and Pereira et al. [12]. PPFP = 60% for anti-*L*. *infantum* IgG1; PPFP = 50% for anti-*T*. *cruzi* IgG1; and PPFP = 60% for anti-*L*. *braziliensis*, respectively. (C) Receiver operating characteristic, ROC curve analyses confirmed the previously selected cut-off and demonstrated outstanding performance indexes: Se, sensitivity; Sp, specificity; PPV, positive predictive value; NPV, negative predictive value; AUC, area under the curve; LR+ and LR–, positive and negative likelihood ratios, respectively, for anti-*L*. *infantum* and anti-*T*. *cruzi* IgG1.

To determine the thresholds for each clinical condition, we titrated the defined patient sera and IgG1 reactivity over the dilution range from 1:1,000 to 1:32,000 (Fig 3A). The reactivity at each dilution was expressed in terms of the PPFP observed for each individual test and against each of the three trypanosomatid species (*L*. *infantum*, *T*. *cruzi*, and *L*. *braziliensis*) in relation to the control of the conjugate (Fig 3A). To determine the thresholds for positive serology, we determined PPFP values for each group of individuals—VL, CH, LCL, and NI (Fig 3B). The best segregation was achieved when the PPFP threshold for positive results for VL was set to 60% at a 1:32,000 dilution. Similarly, the thresholds for CH and LCL were set to 50% and 60% at dilutions of 1:2,000 and 1:1,000, respectively. Such thresholds have also been determined in previously published experiments and for each of the single clinical conditions separately [13, 15]. ROC analyses and evaluation of the performance of each of the parasite preparations and IgG1 reactivity in the different patient groups, as described in Population, Materials, and Methods, demonstrated that PPFP values >60% for *L*.*infantum*, >50% for *T*.*cruzi*, and >60% for *L*. *braziliensis* were the most appropriate cut-offs to distinguish positive results from negative results (PPFP ≤ 60%, ≤50%, and ≤60%, respectively) (Fig 3C).

### FC-Based Inverted Detuned Algorithm for Analysis of Anti-Trypanosomatidae IgG1 Reactivity and Differential Diagnosis of CH and Leishmaniasis

**Fig 4:**
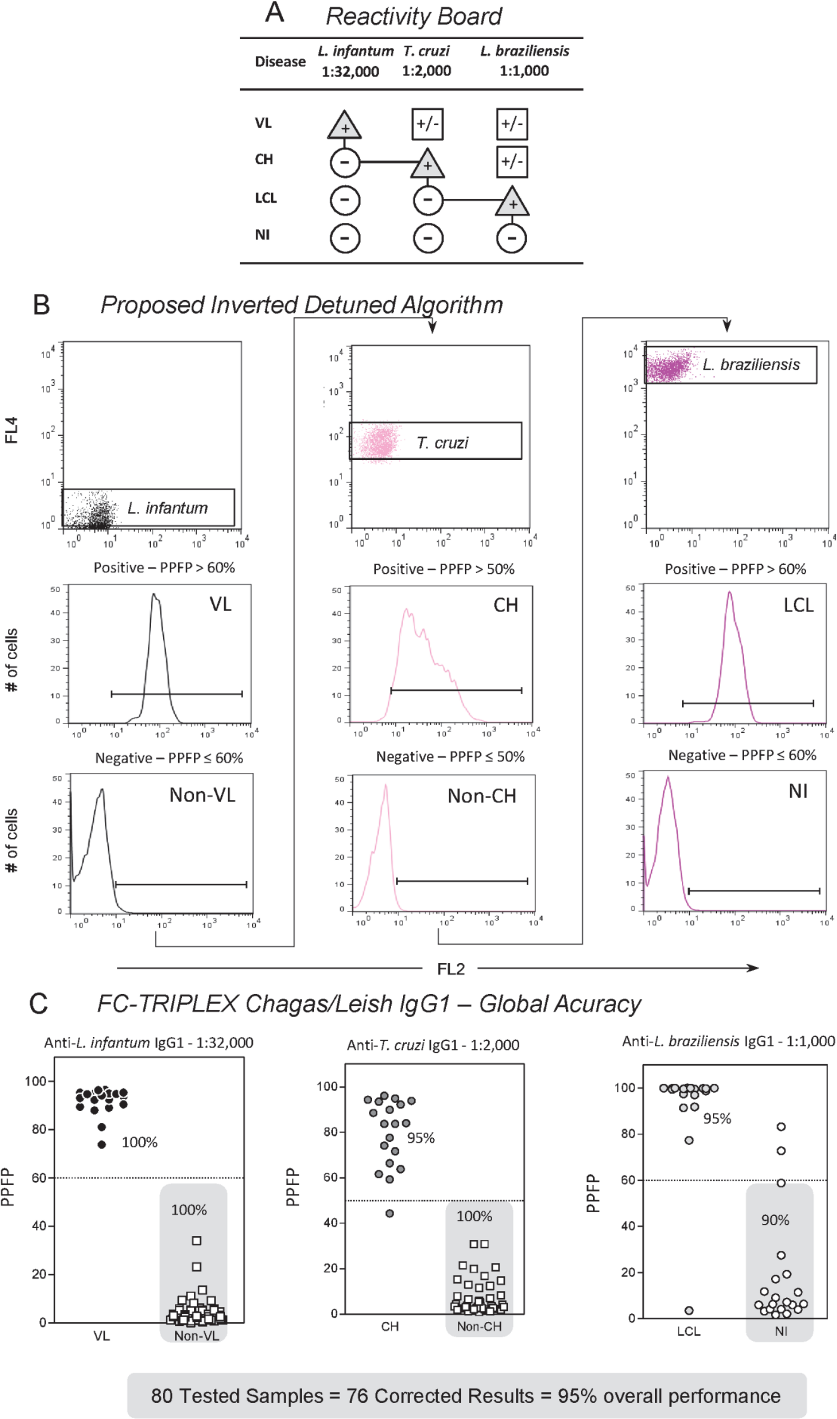
Flow cytometry-based inverted detuned algorithm for analysis of anti-Trypanosomatidae IgG1 reactivity for differential diagnosis of Chagas disease and leishmaniasis. (A) Reactivity board showing the pathway for achieving distinctive disease-related positive results (triangles) and negative results (circles) results and avoiding inconclusive reactivity (squares). VL, visceral leishmaniasis; CH, Chagas disease; LCL, localized cutaneous leishmaniasis; NI, noninfected controls. (B) Proposed inverted detuned algorithm starting at the selection of the *L*. *infantum* population on FL2 versus FL4 bidimensional distribution followed by analyses of IgG1 reactivity at 1:32,000 on unidimensional FL2 histograms expressed as percentage of fluorescence positive parasites (PPFP). A PPFP value of >60% defines the VL diagnosis, whereas a PPFP value of ≤60% leads to the next algorithm step. Selection of the *T*. *cruzi* population is followed by analyses of IgG1 reactivity at 1:2,000, with a PPFP value of >50% defining the CH diagnosis, and a PPFP value of ≤50% leading to the next algorithm stage. At the final step *L*. *braziliensis* population selection is followed by analyses of IgG1 reactivity at 1:1,000, with a PPFP value of >60% defining the LCL diagnosis, and a PPFP value of ≤60% excluding all three Trypanosomatidae infections. (C) Global accuracy analyses underscored the outstanding overall performance of the method, which reached 95% (correct results were obtained for 76 of the 80 serum samples).

For interpretation of serological results, the FC-TRIPLEX Chagas/Leish IgG1 method uses an inverted detuned algorithm to analyze the serologic reactivity of tested samples and to eliminate cross-reactivity for the differential diagnosis of VL, CH, and LCL (Fig 4A and 4B). In this algorithm, the concomitant presence of IgG1 antibodies anti-*L*. *infantum*, anti-*T*. *cruzi*, and anti-*L*. *braziliensis* in a single serum sample was analyzed. The starting point of this algorithm is the selection of the *L*. *infantum* population, on the basis of the FL2 versus FL4 bidimensional distribution, followed by analysis of IgG1 reactivity at 1:32,000 dilution on one-dimensional FL2 histograms and PPFP. A PPFP value of >60% represents a positive result for VL, whereas a PPFP value of ≤60% leads to the next step of the algorithm. Selection of the *T*. *cruzi* population is followed by analysis of IgG1 reactivity at a dilution of 1:2,000, with a PPFP value of >50% resulting in a CH diagnosis, and a PPFP value of ≤50% leading to the next step of the algorithm. Finally, selection of the *L*. *braziliensis* population is followed by analysis of IgG1 reactivity at a 1:1,000 dilution, with a PPFP value of >60% indicating a LCL diagnosis. A PPFP value of ≤60% is considered nonreactive and negative for any of the three trypanosomatid infections.

Results of a pilot study showed excellent performance for the serological differential diagnosis of VL, CH, and LCL. Using 80 serum samples, including negative controls, the flow cytometric method showed an overall performance of 95% (correct results were obtained for 76 of the 80 samples; Fig 4C).

### Influence of Different Lots and Storage Conditions on FC-TRIPLEX Chagas/Leish IgG1 Disease-Related Antigen Recognition

**Fig 5:**
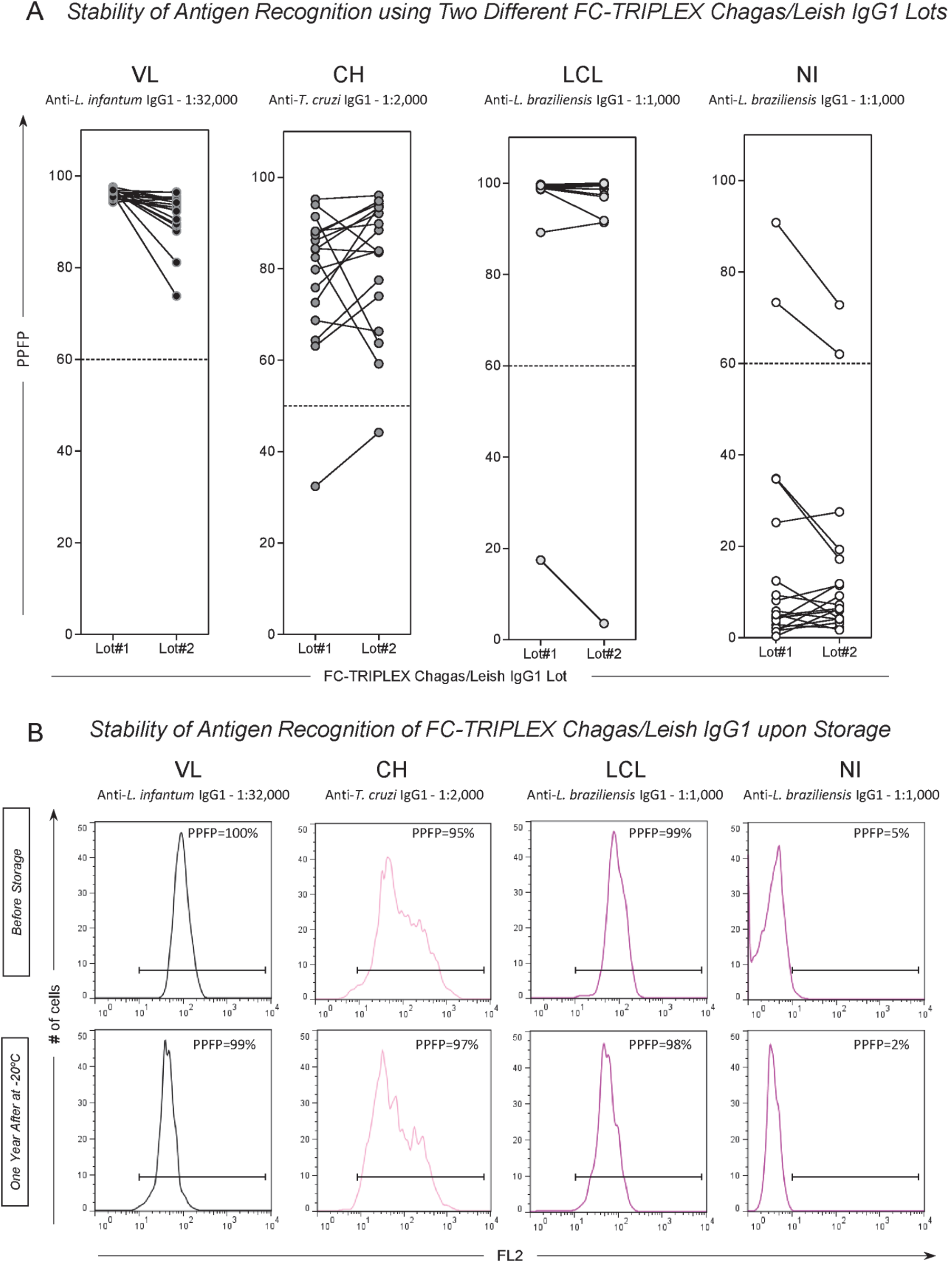
Influence of FC-TRIPLEX Chagas/Leish IgG1 lot and storage on disease-related antigen recognition stability. (A) Two FC-TRIPLEX Chagas/Leish IgG1 lots showed equivalent categorical segregation of anti-Trypanosomatidae IgG1 reactivity at selected serum dilutions (visceral leishmaniasis [VL], anti-*L*. *infantum* at 1:32,000; Chagas disease [CH], anti-*T*. *cruzi* at 1:2,000; localized cutaneous leishmaniasis [LCL] and noninfected controls [NI], anti-*L*. *braziliensis* at 1:1,000). The results are expressed as percentages of fluorescence positive parasites (PPFP) using aligned plot distributions of paired samples. The dotted lines represent the previously determined cut-off edges (PPFP = 60% for anti-*L*. *infantum* IgG1; PPFP = 50% for anti-*T*. *cruzi* IgG1; and PPFP = 60% for anti-*L*. *braziliensis*). (B) Stability of antigen recognition of FC-TRIPLEX Chagas/Leish IgG1 upon storage. Unidimensional FL2 histograms illustrate the similarity of PPFP values obtained using the same lot of FC-TRIPLEX Chagas/Leish IgG1 before storage and after 1 year of storage of parasite suspensions at −20°C. The PPFP values are provided for each histogram.

To verify the reproducibility of antigen recognition in different parasite preparations, two different FC-TRIPLEX Chagas/Leish IgG1 lots were evaluated (Fig 5A). Reproducibility of results was confirmed by equivalent categorical segregation of anti-trypanosomatid IgG1 reactivity at selected serum dilutions (VL, anti-*L*. *infantum* at 1:32,000; CH, anti-*T*. *cruzi* at 1:2,000; LCL and NI, anti-*L*. *braziliensis* at 1:1,000) in both lots (Fig 5A). Finally, the stability of antigen recognition by defined sera of patient groups with the FC-TRIPLEX Chagas/Leish IgG1 method was demonstrated with antigen preparations stored at—20°C for 1 year (Fig 5B).

## Discussion

Serological testing is an essential tool for diagnosing and controlling many infectious diseases [3], and conventional serological tests for CH are routinely used for blood bank screening. However, a non-negative result for CH screening does not necessarily indicate the presence of *T*. *cruzi* infection, because other infectious diseases, such as leishmaniasis, can result in false-positive results. Indeed, various pathogens from the trypanosomatid family (e.g., *Leishmania* spp. and *T*. *cruzi*) share a similar antigenic repertoire with common epitopes that can induce the production of cross-reactive antibodies. Therefore, conventional serological methods usually fail to differentiate infections with these pathogens, especially in areas where leishmaniasis and CH are co-endemic [19]. Furthermore, despite the good performance of commercial kits used in endemic areas, some failures have been reported, mainly owing to the lack of specificity of these kits. Inconclusive results are commonly reported and have been systematically described and analyzed by many investigators, who have reached different conclusions regarding the interpretation of these reactivity patterns [10, 20–21]. One comparison of the performance of different commercial kits for diagnosis of CH revealed that 34.1% of the samples showed inconsistent results in at least one of the tests performed [22]. Discrepancies between the results of serological tests for diagnosis of CH when various tests were performed on the same sample have also been reported in numerous studies [9–10, 22–26]. The variation in the performance of the tests available and the difficulties encountered in the management of inconclusive results highlight the need to implement strategies that minimize or clarify discrepant results. In this context, several studies have been conducted with the goal of developing high-performing confirmatory tests that can be used both in clinical practice and in blood banks, aiming to define with certainty the serological profiles of patients and blood donors [26–28].

Therefore, unconventional serological assays such as the above-described FC-TRIPLEX Chagas/Leish IgG1 method, which includes flow cytometry as a diagnostic tool offer new and interesting possibilities for clarifying inconclusive serological results for neglected tropical diseases. Indeed, owing to its speed, accuracy, and reproducibility, the flow cytometric approach constitutes a major advance in serological assessments of infectious diseases and is becoming increasingly useful both in the clinic and in research laboratories [29].

Previous studies conducted by our group proved the applicability and utility of FC for diagnosis and clinical monitoring of CH and leishmaniasis [12–15, 17, 29, 30]. First, we demonstrated the applicability of FC for serological testing for CH, using live trypomastigote forms of *T*. *cruzi* [17]. In subsequent studies, we verified the applicability and performance of a FC-based method for detecting anti-fixed epimastigote IgG antibodies in sera from individuals infected with *T*. *cruzi* [14, 15]. Additionally, we reported an upgraded FC technology using fixed *Leishmania (Leishmania) amazonensis* promastigotes as antigens for serological diagnosis of cutaneous leishmaniasis [12]. Moreover, we evaluated the performance of an indirect immunofluorescence assay, referred to as FC anti-fixed *L*. *infantum* promastigote IgG antibodies, for serodiagnosis of VL and use of the assay for assessment of post-therapeutic cure [13]. However, despite their good performance and sensitivity, these approaches track only a single disease per test.

To improve our unconventional FC-based approach, we report here, for the first time, the simultaneous detection of anti-*T*. *cruzi*, anti-*L*. *infantum*, and anti-*L*. *braziliensis* IgG1 antibodies, using differential staining of the parasites with FITC or Alexa Fluor 647, on a single flow cytometric platform. The FC-TRIPLEX Chagas/Leish IgG1 method uses the percentage of phycoerythrin-fluorescence-positive parasites as an indicator of seropositivity, and parasite-specific cut-off values were determined. The method is based on the application of an inverted detuned algorithm. Detuned serological approaches have previously been described, and their use to assess post-therapeutic cure in individuals infected with VL has been validated [13, 18]. Our results, obtained with a restricted number of well-defined serum samples, showed 100% sensitivity for VL patients and 95% sensitivity for CH patients and LCL patients. The good performance of FC-TRIPLEX Chagas/Leish IgG1 was confirmed by ROC-curve analysis.

The performance of a diagnostic test is greatly limited by the antigenic preparation used in the test. The use of fixed parasites is a feasible way of storing a bulk amount of antigen and allows for the possibility of producing parasites on a relatively large scale. Moreover, the use of fixed preparations as an antigen source for serological tests eliminates the risk of infection during manipulation of living parasites. In the present work, we evaluated the stability of suspensions of single parasites and mixed parasites, stained either with FITC or with Alexa Fluor 647, under various storage conditions. Our results demonstrated that the suspensions remained stable for at least 1 year; the differential brightness and parasite antigenicity were preserved under the evaluated storage conditions.

We carried out a proof of concept study of the FC-TRIPLEX Chagas/Leish IgG1 method using 80 well-characterized serum samples from individuals with VL, CH, or LCL, and the method provided correct results for 76 of the 80 samples, for an outstanding overall accuracy of 95%. These results show that the method can reliably identify chagasic patients, as well as individuals with VL or LCL.

In summary, we evaluated an unconventional all-in-one FC-based method for differential diagnosis of CH, VL, and LCL. The results obtained by means of this method were highly reproducible, and parasite antigenicity was preserved for an extended period of time under various storage conditions. Owing to the outstanding performance of this method, we propose it as a valuable alternative to conventional serology to elucidate the clinical status of individuals for whom previous tests for CH were inconclusive, especially in leishmaniasis endemic areas. Our data suggest that the method can clarify inconclusive or false-positive results, which are frequently observed in conventional tests used for serological blood bank screening. In future experiments, we will evaluate the performance of the method on a larger panel of sera.
